# Atmospheric Pressure Catalytic Vapor Deposition of Graphene on Liquid Sn and Cu–Sn Alloy Substrates

**DOI:** 10.3390/nano10112150

**Published:** 2020-10-28

**Authors:** Maryam A. Saeed, Ian A. Kinloch, Brian Derby

**Affiliations:** 1 Energy and Building Research Centre, Kuwait Institute for Scientific Research, P.O. Box 24885, Safat 13109, Kuwait; 2 National Graphene Institute, University of Manchester, Oxford Road, Manchester M13 9PL, UK; ian.kinloch@manchester.ac.uk; 3 Department of Materials, University of Manchester, Oxford Road, Manchester M13 9PL, UK; Brian.derby@manchester.ac.uk

**Keywords:** 2D materials, graphene, diffusion and growth, adsorption/desorption, solubility

## Abstract

The chemical vapor deposition (CVD) of graphene on liquid substrates produces high quality graphene films due to the defect-free and atomically flat surfaces of the liquids. Through the detailed study of graphene growth on liquid Sn using atmospheric pressure CVD (APCVD), the quality of graphene has been found to have a close relationship with hydrogen flow rate that reflects on hydrogen partial pressure inside the reactor (P_H2_) and hydrogen solubility of the growth substrates. The role of P_H2_ was found to be crucial, with a low defect density monolayer graphene being obtained in low P_H2_ (90.4 mbar), while partial graphene coverage occurred at high P_H2_ (137.3 mbar). To further understand the role of substrate’s composition, binary alloy with compositions of 20, 30, 50, 60 and 80 wt.% tin in copper were made by arc-melting. Graphene quality was found to decrease with increasing the content of copper in the Cu–Sn alloys when grown using the conditions optimised for Sn substrates and this was related to the change in hydrogen solubility and the high catalytic activity of Cu compared to Sn. This shall provide a tool to help optimising CVD conditions for graphene growth based on the properties of the used catalytic substrate.

## 1. Introduction

Graphene can be synthesised by a variety of methods including mechanical exfoliation [1], liquid phase exfoliation [2,3,4] and chemical vapor deposition (CVD) [5,6,7,8]. Among these methods, the latter shows the most promise for the production of large area, monolayer graphene. The graphene film grown by CVD is found to vary in quality, thickness and morphology depending on both the growth substrate and conditions used. The catalytic substrate plays a key role in the CVD process kinetics, defining the choice of the growth temperature (high/low). [9] A wide range of precious and transition metals have been used as substrates, including copper (Cu) [7,10,11,12], nickel (Ni) [13,14,15], platinum (Pt), ruthenium (Ru) [16,17], cobalt (Co) [18], rhenium (Re) [19] and palladium (Pd) [20]. A key parameter in the choice of substrate, and its corresponding growth conditions, is the carbon solubility in the substrate, which is metal dependent and defines the graphene deposition mechanism [15,21]. In low carbon solubility metals such as Cu, the deposition mechanism is governed by surface adsorption and diffusion of the active carbon species [7,15,22], whereas in high carbon solubility metals, such as in Ni substrates, the growth mechanism is mainly by bulk diffusion of the active carbon species followed by surface precipitation during cooling step [15,22,23]. Typically, the growth substrates are used in their solid phase, which means their morphology (e.g., surface roughness and grain boundaries) strongly affect the quality of the graphene grown. For example, carbon atoms prefer to precipitate on regions of the substrate with higher energy such as impurities and grain boundaries [24]. Thus, such substrate defects mean that the resulting graphene film is not uniform in thickness, with multi-layer graphene found around the grain boundaries and other surface defects, while randomly orientated thin sheets are found in the other regions. One route to overcome this problem is to use liquid metal substrates. In the absence of any surface oxide or contamination, a liquid metal surface is levelled by gravitational force, has a uniform local atomic structure and contains no topological or crystal defects. There have been a number of reports in the literature of graphene films being grown on Cu substrates held above their melting temperature [12,25,26,27,28,29]. It has been reported that the nature and the properties of the produced graphene on liquid Cu is sensitive to the CVD growth parameters. Furthermore, more liquid metals were used to grow graphene via CVD method such as gallium (Ga), indium (In) and tin (Sn) [30,31].

In this report, we studied the effect of various process conditions on the growth of CVD graphene on liquid Sn, highlighting the role of hydrogen. In order to optimise the process conditions, a detailed investigation was conducted to study the effect of residence time of the reactive species. This was a starting point to optimise the total gas flow rate and carbon concentration. The catalytic activity of Cu and the change in physical/chemical properties of the growth substrate were explored by using different compositions of Cu–Sn alloys. Such a study has never been reported before; thus, this should provide new insight on the role of hydrogen solubility of the growth substrate in determining the optimum hydrogen flow rate/partial pressure and hydrocarbon concentration. Furthermore, process temperature was investigated showing its effect on the decomposition of CH_4_ and its effect on the substrate’s hydrogen solubility. This systematic study shed the light on the correlation between the properties of the substrate used and the effect of the various process conditions.

## 2. Materials and Methods

Pure Sn (99.995%) and Cu (99.9999%) metals were used (Goodfellow Cambridge Ltd., Huntingdon, UK) as growth substrates, held in tungsten boats (Agar Scientific, Elektron Technology UK Ltd., Stansted, UK). Cu–Sn alloys were prepared using an arc-melting technique; elemental pure Cu and Sn were selected as to give a total weight of 5 g at various nominal compositions (20, 30, 50, 60 and 80 of Sn wt.%) with their compositions and melting points given in Supplementary Materials Table S1.

In a typical APCVD run, a sample was loaded at the centre of the isothermic zone and the reaction tube was first evacuated to base pressure then backfilled with hydrogen/argon mixture until atmospheric pressure was reached. The furnace then was switched on to gradually heat and reach the desired temperature. Then, the sample was left for annealing at that temperature for 30 min. The growth step began by introducing the hydrocarbon feedstock (methane) for the desired growth time. Finally, methane and the furnace were switched off and the chamber was left to cool naturally to room temperature under flow of hydrogen/argon mixture. Each CVD run was typically repeated 3–5 times to ensure consistency. CVD reaction tube was purged and filled with nitrogen after each run to ensure clean environment.

Field emission gun scanning electron microscopy (FEG-SEM) (FEGSEM, FEI, Eindhoven, The Netherlands) was used for the characterisation of the as-grown graphene. An accelerating voltage of 8 kV was used with a maximum working distance of 10 mm.

Raman spectroscopy was the predominant technique used to study the graphene grown on the different substrates. All Raman measurements were carried out using a Renishaw inVia system with an excitation laser wavelength of 633 nm (Renishaw, Wooton-under-Edge, UK). The main graphene characteristic peaks (D, G and 2D peaks) Raman peaks were fitted using mixed Lorentzian–Gaussian functions in the Wire 4.2^TM^ software (Renishaw, Wooton-under-Edge, UK). The software was used to calculate the band intensity ratio, I_2D_/I_G_ and I_D_/I_G_.

## 3. Results and Discussion

### 3.1. CVD Graphene Growth on Liquid Sn Substrates—The Study of Hydrogen Effect and Temperature Control

The CVD process requires careful tuning of the growth conditions based on the properties of the substrate used (e.g., carbon solubility) as in the case of graphene deposition. The process-limiting phenomena can be either surface-reaction-rate or gas-phase limitations [32]. The system used for this study is APCVD; thus, the carbon transport is mass limited in gas phase unlike the surface limited growth for LPCVD [33]. Gas phase transport limitations are controlled by gas-related conditions such as the concentration of reactive species, residence time and flow rate. Therefore, the growth conditions are studied thoroughly with correlation to the substrates’ hydrogen solubility.

#### 3.1.1. The Effect of Residence Time Modulated by Hydrogen/Methane Ratio

Studying the residence time, τ, of the reactive gases can elucidate the reaction equilibrium within the reactor and how it effects the subsequent graphene deposition. It could be described as the time that the reactants spend in the gas stream in the CVD reactor with a laminar flow rate, and it is inversely proportional to the total gaseous mass flow rate [34]. Lewis et al. calculated the residence time by dividing the length of the isothermal zone of the reactor, L_iso_, by the mean flow velocity, <ν> [35]. The ideal gas model and the principal of conservation of mass were used to convert the volumetric flow rate at which gases are introduced to the chamber (F_s_) to that within the chamber (F_r_). <ν> was then calculated by dividing the cross-sectional reaction tube. Thus, <*ν*> in terms of the reaction pressure P_r_, and temperature T_r_, was given by

(1) $$\langle \nu \rangle =\frac{F_{r}}{{\pi r}^{2}}=\frac{F_{s}P_{s}T_{r}}{T_{s}P_{r}{\pi r}^{2}}$$

F_s_ was measured at standard temperature and pressure, (T_s_ = 293.15 K and P_s_ = 101,325 Pa). Thus, τ was calculated by Equation (2):

(2) $$\tau =\;\frac{L_{\mathrm{iso}}T_{s}P_{r}{\pi r}^{2}}{F_{s}P_{s}T_{r}}$$

The residence time (RT) was investigated in combination with increasing the methane ratio (R_CH4_) in the reactor by reducing the H_2_ and Ar flow rates and keeping the CH_4_ flow constant at 5 sccm. Different H_2_ and Ar flow rates were used in order to change the total flow rate in the reaction chamber (Table S2).

Raman mapping were collected from 121 points (11 × 11 points, 22 µm) for each of the as-grown graphene films on the ex-situ, solidified Sn substrate. These spectra were then used to map the band intensity ratios I_2D_/I_G_ and I_D_/I_G_. The 2D band was a symmetric single Lorentzian line shape with a full width at half-maximum (fwhm) of <36.2 cm^−1^ for samples grown using 25 H_2_:250 Ar:5 CH_4_ (sccm), giving CH_4_ ratio (R_CH4_) = 0.017 and RT = 12.8 s. These samples also had an average Raman intensity ratio I_2D_/I_G_ of 1.6, which is characteristic of single layer graphene and a I_D_/I_G_ of 0.34 which indicated low defect density in graphene with a low defect density and large graphene domain.

**Figure 1:**
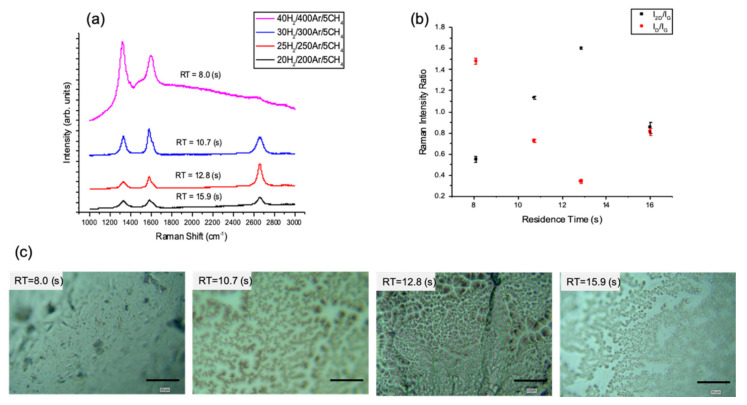
(**a**) Typical Raman spectra of the APCVD graphene grown on liquid Sn for different residence times (RTs) and H_2_:CH_4_ ratios. (**b**) Average Raman peaks intensity ratios I_2D_/I_G_ and I_D_/I_G_ with respect to residence time. (Error bars are smaller than some data points.) (**c**) Optical images of the grown graphene films (scale bar: 20 µm), some blurriness is due to the surface curvature of the solidified Sn after melting. (APCVD, T = 1120 °C, (20–40) H_2_/(200–400) Ar/5 CH_4_ sccm, t = 5 min).

In Figure 1b, the average Raman peaks intensity ratios show a clear dependence on R_CH4_ and RT. The average I_2D_/I_G_ increases while I_D_/I_G_ decreases with the increase of RT while the coverage is full at RT = 12.8 (Figure 1c) until it reaches a critical point of mass transport limiting the deposition. A hypothesis for the observed results is that the high R_CH4_ of 0.22 and long RT of 15.99 are considered critically high values for the process that led to saturation of the reactant species in the gas stream and on the surface of the substrate, thus increasing the nucleation rate. This increased rate that eventually caused the deposition of few layer graphene and decreased domain size while surface coverage is disrupted. In comparison, at lower R_CH4_ of 0.002 and RT of 8 s, the Raman spectrum (Figure 1a) do not feature any graphene peaks.

#### 3.1.2. The Effect of Hydrogen Flow Rate

The effect of H_2_ flow rate and consequent changes to partial pressure on graphene growth remains unexplored for the case of liquid Sn substrates. In order to investigate the influence of hydrogen on the nature of the deposited graphene film and the way it influences growth kinetics, different H_2_ partial pressures (P_H2_) were obtained by adjusting the H_2_ flow rate during the APCVD run. This P_H2_ was kept constant throughout the experiment (i.e., annealing, growth and cooling). All the remaining growth parameters (temperature, Ar and CH_4_ flow rates and growth time) were kept constant throughout this experiment series (Table S3).

**Figure 2:**
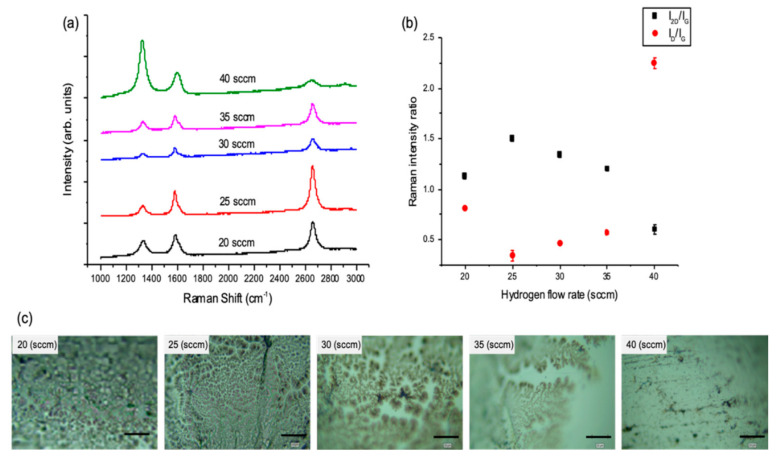
(**a**) Raman spectra of graphene grown on liquid Sn showing the effect of changing the H_2_ flow rate. (**b**) Average Raman intensity ratio I_2D_/I_G_ and I_D_/I_G_ values vs. the H_2_ flow rate based on maps data. (Error bars are smaller than some data points.) (**c**) Optical images of the grown graphene films (scale bar: 20 µm), some blurriness is due to the surface curvature of the solidified Sn after melting (APCVD, T = 1120 °C, (20–40) H_2_/250 Ar/5 CH_4_ sccm, t = 5 min).

Optical microscopy and Raman spectroscopy found that at the formation of graphene film was disrupted at the highest H_2_ flow rate and instead amorphous carbon was deposited (Figure 2). This observation is in agreement with the reported literature where the conversion rate of CH_4_ is inhibited in the presence of a rich hydrogen environment due to the depression of the CH_4_ dehydrogenation [36]. Raman spectroscopy found little variation in the I_2D_/I_G_ and I_D_/I_G_ ratios at the three intermediate H_2_ flow rates used (25, 30 and 35 sccm) (Figure 2a,b) based on points collected in the Raman maps. However, there was a major difference in these graphene films under optical images (Figure 2c), where the graphene coverage is more complete at the lower H_2_ flow rates (best at 20 sccm). The lowest H_2_ flow rate, 20 sccm, was considered as a critical point for the flow rate. This leads to a rich hydrocarbon environment causing saturation of reactive species and an increase in the number of deposited graphene nuclei, a smaller grain size and a more intense D band in the Raman spectrum, which indicates a decrease in graphene quality. These results showing the change in film morphology and defect density as a function of H_2_ flow rate indicates that the use of lower H_2_ flow rates (excluding its minimum critical point) can improve the quality of graphene on liquid Sn probably as it can transform sp^3^ bonds into sp^2^. [37] This observation can also be explained by the low hydrogen solubility in Sn. Losurdo et al. studied the growth of graphene on Ni and Cu, noting that Ni has a lower hydrogen solubility and diffusivity than in Cu. They monitored graphene deposition in real-time by recording variations of the Cu and Ni dielectric function during exposure to H_2_. [15] They found that the hydrogen diffused 15 nm deep into the Cu compared to 12 nm in the Ni. Furthermore, H_2_ diffused out of the Cu when the H_2_ flow was shut-off, whereas no reversible response was measured in Ni, suggesting that H_2_ desorbed from the Ni surface. It was found that by optimising and minimising hydrogen content in Ni substrate, graphene without a Raman D-peak could be achieved. However, this role of H_2_ substrate solubility on the CVD of graphene has not been confirmed with other substrates such as Sn. Indeed, similar to Ni, the hydrogen in Sn is low compared to Cu [38]; thus, hydrogen dissolves into the bulk of the Cu substrate during the annealing step and then diffuses out to the surface to promote graphene nucleation during growth [39]. However, in Sn, it is expected that the H_2_ interactions are surface limited due to the poor bulk solubility. Therefore, lower hydrogen flow rate is more favourable in the case on Sn growth substrate as demonstrated and confirmed by the obtained results.

#### 3.1.3. The Effect of Reactant Species (H_2_ and CH_4_) Partial Pressure

**Figure 3:**
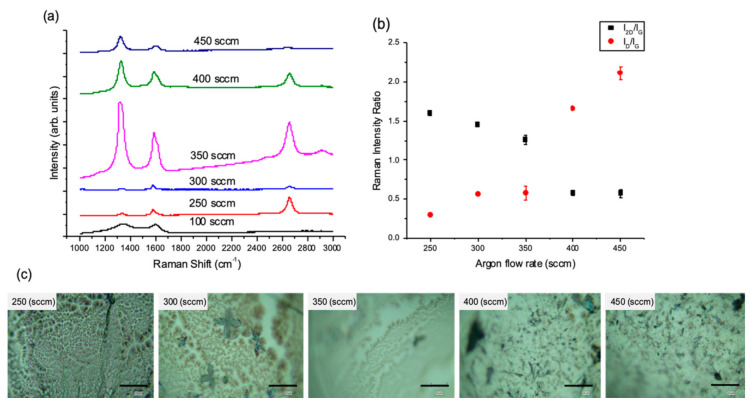
(**a**) Raman spectra of graphene grown on liquid Sn showing the effect of changing the Ar flow rate. (**b**) Average Raman intensity ratio I_2D_/I_G_ and I_D_/I_G_ values vs. the Ar flow rate based on maps data. (Error bars are smaller than some data points). (**c**) Optical images of the grown graphene films (scale bar: 20 µm), some blurriness is due to the surface curvature of the solidified Sn after melting (APCVD, T = 1120 °C, 25 H_2_/(100–450) Ar/5 CH_4_ sccm, t = 5 min).

The total partial pressure of the reactant components (*P_H2+CH4_*) in the reactor can be controlled through either the total pressure in the reaction chamber *P_t_* or the flow rate of a diluent gas such as Ar(_g_) or N_2(g)_. Thus, different Ar flow rates were introduced into the reaction chamber to tune the *P_H2+CH4_* (Table S4). The degree of graphene coverage as a function of *P_H2+CH4_* can be seen on the optical microscope images in Figure 3c. Maps of the Raman intensity ratios were conducted, their average values plotted in Figure 3b and representative single Raman spectra in Figure 3a. Full graphene coverage was achieved at higher *P_H2+CH4_* when the Ar flow rate was set to 250 sccm. The intermediate Ar flow rates, 300 and 350 sccm, resulted in partial graphene coverage on the Sn surface, while the highest flow rates, 400 and 450 sccm, resulted in sooty carbon depositions.

**Figure 4:**
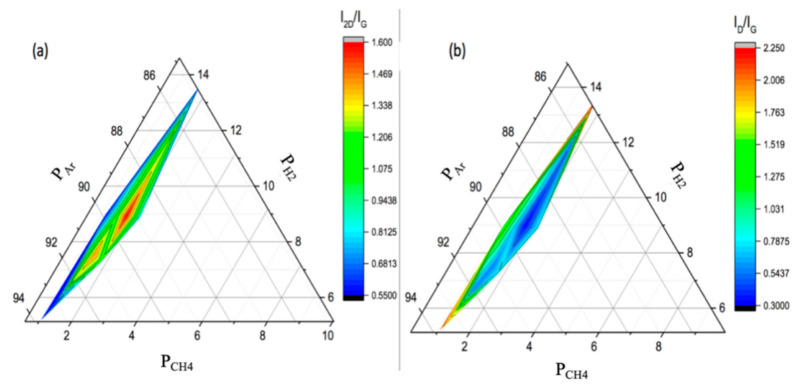
Ternary plots showing Raman intensity ratios as a function of gas partial pressures (P_H2_, P_Ar_ and P_CH4_), (**a**) I_2D_/I_G_ and (**b**) I_D_/I_G_ (All axes are normalised with respect to total gas pressure.).

It was found that the best quality graphene was grown at *P_H2+CH4_* = 108.4 mbar when using an Ar flow rate of 250 sccm (as identified by Raman spectroscopy). Lowering the Ar flow rate to 100 sccm, i.e., increasing the *P_H2+CH4_* to 233.76 mbar, caused the deposition of amorphous carbon. While reducing *P_H2+CH4_* via increasing the Ar flow rates (250–450 sccm) led to an increasing number of graphene layers to be deposited, as indicated by the average Raman intensity ratio I_2D_/I_G_ (Figure 3b). In contrast, the I_D_/I_G_ ratio in Figure 3b shows two regimes: (1) high Ar flow rates where I_D_/I_G_ increases proportionally with flow rate, indicating a higher density of defects, and (ii) low Ar flow rates (100 sccm) when I_D_/I_G_ begins to decrease due to the increasing amorphous nature of the deposited films [40]. The increase in Ar flow rate affects the deposition kinetics as it is assumed to cause an increased collision rate between the active gas molecules and the substrate surface [41]. Therefore, the high carbon solubility in Sn combined with the increased concentration of the active radicals on the substrate lead to an increase in the thickness of the deposited graphene. In order to summarise the results, ternary plots were generated, where each axis represents the partial pressure of each gas, CH_4_, H_2_ and Ar. Thus, each axis goes from 0 to the reaction pressure, as the total pressure is constant for all systems. The Raman intensity ratios, I_2D_/I_G_ and I_D_/I_G_ are plotted as ternary contour plots for visualisation in Figure 4.

In the ternary plots (Figure 4), it can be seen that better graphene quality, according to the Raman intensity ratios, occurs at high carbon concentration. The high I_2D_/I_G_ and low density of defects I_D_/I_G_, which are associated with considerably high-quality graphene, are found at the higher partial pressures of CH_4_ and H_2_. In the case of Sn, it was found that in order to reach full coverage of monolayer graphene with a low density of defects, a high carbon concentration was required, which is opposite to the observations for APCVD graphene grown on Cu as reported in the literature [42,43]. This suggests that the graphene growth mechanism in Sn happens through segregation, when carbon atoms are diffused in the bulk and the graphene is formed during cooling, with carbon atoms precipitated to the surface. This may also explain the observed improvement in graphene coverage at higher carbon concentrations that lead to a uniform carbon saturation in the bulk, resulting in uniform carbon precipitation.

#### 3.1.4. Effect of Growth Temperature

**Figure 5:**
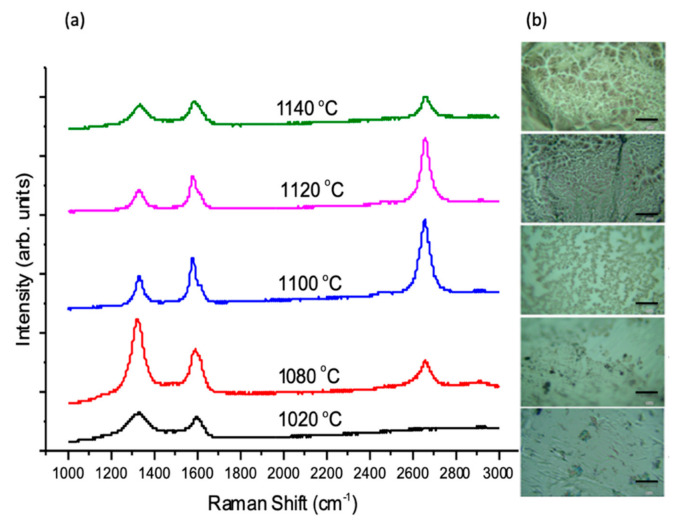
(**a**) Raman spectra of graphene grown on liquid Sn at different growth temperatures on liquid Sn using the optimised growth conditions on Sn (APCVD, 25 H_2_/250 Ar/5CH_4_ sccm, t = 5 min.). (**b**) Optical images of the deposited graphene film (scale bar 20 µm); some blurriness is due to the surface curvature of the solidified Sn after melting.

Another crucial parameter investigated was growth temperature. Initial experiments of graphene growth on liquid Sn used a growth temperature of T = 1020 °C. This temperature was selected because it had been extensively reported in the literature as successful for the growth of graphene on Cu and had also been reported with work by other groups on liquid Sn substrates [10,31]. The temperature affects not only reaction rate and equilibrium gas composition, but also nucleation and, therefore, the microstructure of the deposited film. Low temperatures have been found to led to fine-grained graphene sheets, as they decrease the diffusion length of the dissociated carbon ad-atoms after the reaction according to the surface kinetics [44,45]. Moreover, higher temperatures were reported to lead to an increased desorption rate which consequently decrease the nucleation density, leading to larger graphene domains [46,47]. Different growth temperatures were explored for the liquid Sn substrates while keeping all other parameters as constant (e.g., growth time, pressure and gases flow rates). Figure 5 shows the clear enhancement of graphene quality and coverage with increasing growth temperature.

**Figure 6:**
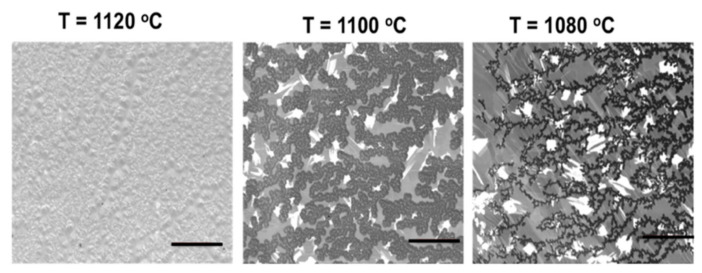
SEM micrographs of deposited graphene on liquid Sn showing the evolution of the graphene coverage due to the increase in growth temperature. Scale bar: 20 μm.

The graphene grown at T = 1100 °C and T = 1120 °C showed graphene films that exhibit high I_2D_/I_G_ (≈1.4–1.7) in agreement with monolayer graphene. Moreover, both have relatively low I_D_/I_G_ (≈0.3–0.5) indicating low defect density, with the D’ band present as a right shoulder on the G peak. D’ is not widely considered due its small intensity in comparison to the D peak. However, it is clearly distinguished from the G peak in the case of the two growth temperatures used (1100 and 1120 °C), indicating a moderate density of defects [48]. Although both temperatures exhibit similar effects on the deposition with a small difference of 20 °C, the graphene coverage obviously differs. The optical images shown in Figure 5b show branch-like depositions at T = 1100 °C with areas of uncovered bare metal, while full coverage has been obtained at T = 1120 °C. This observation was confirmed in the SEM, which show the evolution of graphene coverage at growth temperatures of 1080 °C, 1100 °C and 1120 °C (Figure 6). Moreover, it was found that by further reducing the growth temperature, an amorphous carbon is obtained. This poor growth at lower temperatures is disappointing in that an obvious benefit of Sn is its low melting point and hence the possibility to use it as a low temperature growth substrate.

The temperature effect on deposition can be explained by considering that as temperature increases, a greater proportion of the adsorbed carbon can surmount the energy barrier, achieving attachment and contributing to graphene formation [46]. Moreover, a higher film growth rate is considered to be an important reason for the observation of graphene full coverage at higher temperatures. It would have been interesting to try higher temperatures; however, the softening point for the quartz reaction tube was 1150 °C, limiting the current work to a maximum temperature of 1140 °C.

The highest growth temperature used was 1140 °C, which gave Raman spectra with a relatively high intensity ratio of I_D_/I_G_ ≈ 1, indicating that this is due to phonon scattering from in-plane defects (D band), rather than from stacking induced disorder [49,50,51]. Moreover, the intensity ratio I_2D_/I_G_ ≈ 0.8–1 is characteristic of bilayer to few layer graphene deposition. Another observation is the changed appearance of the quartz reactor tube, which turned black towards the end of the exhaust end during the reaction (Figure S1).

The increase in hydrogen solubility at elevated temperature can be explained by Sievert’s law (Equation (3)):

(3) $$C_{H}=\sqrt{\frac{P_{H_{2}}}{P_{0}}}\exp\left[-\frac{\Delta G_{m}^{*}}{\mathrm{RT}}\right]$$

where C_H_ is hydrogen solubility, $G_{m}^{*}$ (J/mol) is the change of molar free energy of hydrogen during solution, $P_{H_{2}}$(Pa) is the pressure of hydrogen on the liquid metal/alloy, P_0_ is the standard pressure, R is molar gas constant and T(K) is the temperature [52]. A growth temperature of 1140 °C led to increased hydrogen solubility. This caused more hydrogen desorption into the bulk of Sn and its aggregation to the surface providing more active sites leading to increased number of graphene layers. More detailed discussion on the effect of increased hydrogen solubility is reported in the following section of using Cu–Sn alloys.

Generally, the results obtained using different growth temperatures confirm that elevated temperatures increase the rate of CH_4_ decomposition, causing the gaseous composition to change due to greater thermodynamic stability of larger hydrocarbons at higher temperatures. [53].

Although the produced graphene films exhibited varying D peak intensities, the demonstrated optimal conditions showed low defects density. The conducted experiments and results should provide an insight on the effect of the used substrates’ properties on determining the optimum CVD conditions in order to tune the characteristics of the produced graphene film. This should pave the way to explore possibilities for simple and direct graphene transfer from the molten metal.

### 3.2. CVD Graphene Growth on Liquid Cu–Sn Alloys—The Study of Catalytic Activity of Copper

As aforementioned, the ideal growth conditions for graphene deposition between Cu and Sn are very different; Cu needs higher hydrogen flow rate, while Sn requires lower hydrogen flow rate. Moreover, a Cu–Sn alloy has a higher carbon solubility than bare Cu substrate [54,55]; therefore, a change in substrates’ properties will be obtained by varying the Cu–Sn alloy composition. In order to understand the change in graphene deposition between Cu and Sn, five compositions of Cu–Sn binary alloys were used as growth substrates for the formation of APCVD graphene. Experiments were conducted using the optimised parameters for pure liquid Sn (Table S5), while EDX elemental analysis for the growth alloy substrates was conducted after the CVD run to compare and ensure that the alloy compositions have not drastically changed due evaporation (Table S6).

**Figure 7:**
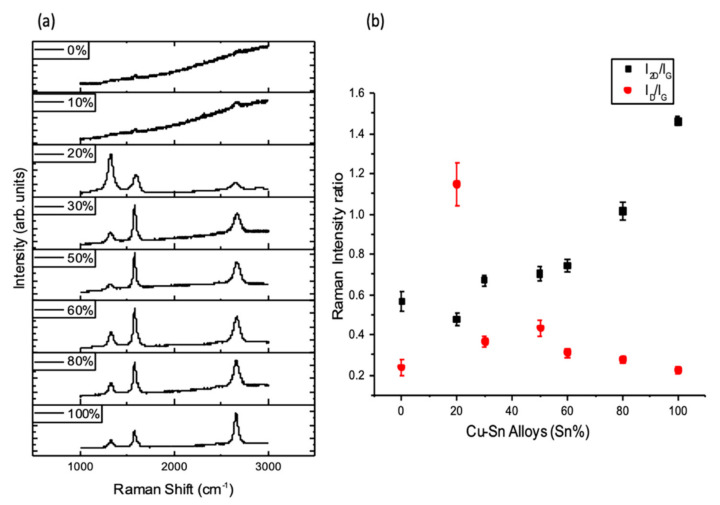
(**a**) Raman spectra of points taken randomly on the surface of the deposited film for the different Cu–Sn compositions (Sn%). (**b**) A plot of the average Raman intensity ratios based on >100 points Raman maps, I_2D_/I_G_ and I_D_/I_G_ vs. the wt.% content of Sn in each Cu–Sn composition (error bars are smaller than some data points) (APCVD, T = 1120 °C, 25 H_2_/250 Ar/5 CH_4_ sccm, t = 5 min).

Raman mapping was taken on the as grown graphene film on each substrate, and Raman spectra representing the resulted graphene on each Cu–Sn composition are shown in Figure 7a, while average Raman intensity ratio I_2D_/I_G_ and I_D_/I_G_ of the samples, demonstrated in Figure 7b.

The number of graphene layers was found to increase with the high Cu content in the alloy composition. When Cu wt.% is in the range of 80 to 100%, an obvious degradation in graphene quality occurs under the ideal growth conditions for Sn, with the D band activated due to the high presence of defects in Cu_80%_–Sn_20%_. The proposed scenario of an increased number of graphene layers as the content of Cu in the alloy increases, is believed to be due to the catalytic nature of Cu. As Cu catalytically stimulates CH_4_ cracking, higher Cu wt.% leads to a faster and higher CH_4_ decomposition rate, causing accelerated graphene nucleation and deposition. [56].

**Figure 8:**
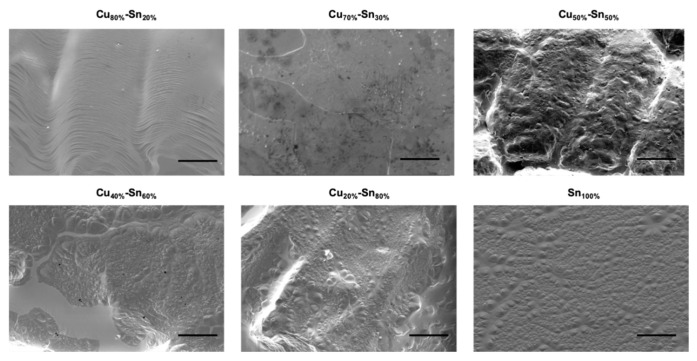
SEM images of Cu–Sn alloys at different compositions after APCVD graphene growth (Scale bar: 20 µm).

SEM micrographs of the deposited graphene films found a uniform image contrast over the semi-flat surface for pure Sn (Figure 8), indicating the deposition of a uniform graphene film. However, not having a completely flat surface as that obtained on liquid Cu may be due the surface contraction of liquid Sn via solidification. Despite the surface topography, the deposited graphene on liquid Sn exhibited monolayer and a high-quality nature as confirmed by Raman mapping. Introducing Cu into the alloy at small concentration of Cu_20%_–Sn_80%_ resulted in a bumpy surface, while increasing Cu further to 40% resulted in the formation of big cracks causing partial graphene coverage. At 80% Cu, the SEM image showed a wavy surface, the propagation of this surface waves of the deposited graphitic film onto the Cu-rich alloy surface was most likely due to the wetting of the growing graphitic material with the liquid Cu layer. Voids are also seen within the propagating waves, leading to areas with no graphene deposition.

Another possible scenario for the change in graphene layers with the various Cu content in the Cu–Sn alloys is due to the change in H_2_ solubility for each of the different compositions (Figure S2). As mentioned above, the solubility of H_2_ in liquid Cu is greater than in liquid Sn. H_2_ solubility in liquid Cu and several liquid binary Cu alloys has been measured using the Sieverts method. It was found that alloying elements, Co, Fe, Mn and Te, increased solubility while Sn, Zn, S, P and Sb decreased it [38]. Therefore, by increasing the Cu content in the Cu–Sn alloy, H_2_ solubility increased [38]; thus, the H_2_ diffusivity in the molten metal was affected. This implies that diffusion rate of hydrogen molecules into the bulk of the substrate was enhanced by the content of Cu. The increasing presence of H_2_ molecules in the subsurface of the Cu–Sn substrate can lead to two possible behaviours: The first one is where hydrogen molecules may diffuse out to the surface and cause undesired bonds with carbon atoms from the graphene film, desorbing as volatile compounds [39]. The second possibility, although still unlikely, is that the diffused H_2_ molecules in the bulk may react with impurities such as oxides that lead to the production of water vapor, creating pressure in the metal and causing a phenomenon called “hydrogen embrittlement”. The degassing of H_2_O molecules in any stage of the growth process (growth/cooling) creates cracks in the graphene film, leaving bare metal which may describe the breakage in the graphene film [39] when Cu-rich compositions were used.

## 4. Conclusions

In conclusion, APCVD graphene grown on liquid Sn and liquid Cu–Sn alloys has been demonstrated as a potential avenue for large-area continuous graphene films. Using APCVD to grow graphene on liquid Sn, the experiments have shown the graphene quality and the growth kinetics are both sensitive to reactants’ residence time, hydrogen flow rate, hydrocarbon partial pressure and growth temperature. When considering CVD growth of graphene on liquid Cu–Sn alloy at different compositions, important factors have been suggested to include their relative catalytic abilities, carbon solubility and hydrogen solubility. It has been demonstrated that graphene quality decreases with increasing the content of Cu in the Cu–Sn alloys when grown using the conditions optimised for Sn substrates. This can be explained due to the change in hydrogen solubility in the Cu–Sn compositions and also because of the different catalytic nature of Cu, leading to faster CH_4_ decomposition (i.e., the growth environment turns into active carbon radical rich one). This study not only provides an alternative method to grow large-area graphene films on liquid catalytic substrates, but also points out more important factors, such as hydrogen and carbon solubility and hydrogen partial pressure, to influence the quality of graphene.

## Supplementary Materials

The following are available online at https://www.mdpi.com/2079-4991/10/11/2150/s1, Table S1: The weight of elemental metal used to form each Cu–Sn alloy composition, Table S2: Summary of the used APCVD graphene growth parameters on liquid Sn to study the effect of CH_4_ concentration and residence time, Table S3: APCVD graphene growth parameters on liquid Sn using different H_2_ flow rates, Table S4: APCVD graphene growth parameters on liquid Sn using different Ar flow rates, Figure S1: Photograph of the CVD reaction tube showing the change in colour of the tube’s inner walls turning to black due to saturation of carbon species after using growth temperature 1140 °C for 5 min, Table S5: Optimised growth parameters for APCVD graphene on liquid Sn, Table S6: EDX compositional analysis of samples after APCVD graphene growth, in order to make comparisons with the initial compositions, Figure S2: Solubility of hydrogen in liquid Cu–Sn alloys as a function of Sn wt.% at 1100 °C.
