# Supplementary material for: Atmospheric Pressure Catalytic Vapor Deposition of Graphene on Liquid Sn and Cu–Sn Alloy Substrates
Source: Nanomaterials (Basel). 2020 Oct 28;10(11):2150. doi: 10.3390/nano10112150 (PMC7692589; doi:10.3390/nano10112150)
Supplement: Supplementary file 1 [file nanomaterials-10-02150-s001.pdf]

Supplementary Materials

Atmospheric Pressure Catalytic Vapor Deposition of Graphene on Liquid Sn and Cu–Sn Alloy Substrates

Maryam A. Saeed [1,2,3,*], Ian A. Kinloch [2,3] and Brian Derby [3]

[1] Energy and Building Research Centre, Kuwait Institute for Scientific Research, P.O. Box 24885, Safat 13109, Kuwait
[2] National Graphene Institute, University of Manchester, Oxford Road, Manchester M13 9PL, UK; ian.kinloch@manchester.ac.uk
[3] Department of Materials, University of Manchester, Oxford Road, Manchester M13 9PL, UK; Brian.derby@manchester.ac.uk
* Correspondence: msaeed@kisr.edu.kw; Tel.: +965-99490373

APCVD setup and graphene growth

All APCVD runs were conducted using a 1.5 m long quartz tube (GE214A, Multi-lab Quartz & Ceramic Technology) placed in a 3-zone furnace (0.75 m heat zone, PSC 12/90/600H Lenton, Hope, UK). A gas control unit was used to deliver hydrogen (99.95%), methane (99.5 %) as the hydrocarbon feedstock, argon (BOC 99.98%) and nitrogen (99.998%). A rotary vane pump (Edwards RV12) was used to evacuate the reaction tube and a one-way pressure relief valve (PRV) was connected to maintain atmospheric pressure in the tube during the APCVD process.

In a typical reaction, the rotary vane pump was first switched on and the manual valve was gradually opened to evacuate the reaction chamber. After reaching a pressure of ≈2.3×10^{-3} mbar, hydrogen/argon mixture was introduced into the reactor at the desired flow rate. Next, the manual valve was closed and the rotary vane pump switched off to allow the pressure to build up to atmospheric in the reaction chamber. After the pressure in the reactor reached ≈1 bar, the pressure relief valve was opened automatically to allow the passage of excess gas and to maintain atmospheric pressure in the reactor. Once atmospheric pressure was achieved, the furnace was switched on and set to a temperature above the melting point of the substrate in the boat; in most cases, the growth temperature was set to 1120 °C. After the furnace had reached the growth temperature, it was held for 30 min under a flow of a mixture of hydrogen and argon. The growth step began when the hydrocarbon feedstock (methane) was introduced into the reaction chamber. After the required reaction time, the methane flow was switched off and the chamber was left to cool naturally to room temperature under flow of hydrogen/argon mixture. Each CVD run was typically repeated 3–5 times to ensure consistency. CVD reaction tube was purged and filled with nitrogen after each run to ensure clean environment.

Cu–Sn Alloy: Compositions/preparation

Pure metals Sn and Cu, with purity 99.995% and 99.9999% with a maximum lump size of 4 mm and 5 mm respectively, all metals being used as-received. The substrates (Pure Sn or Cu–Sn alloy) were held in tungsten boats (AGE439, 32 mm × 0.05 mm).

The Cu and Sn pellets were placed in the hearth with eight rooms that allowed the production of five button-shape alloys of different compositions in the same run. The melting process, which is achieved under an argon gas atmosphere (99.999%), started with the melting of a pure zirconium (Zr) getter to

remove the residual oxygen from the chamber of the arc melter. To ensure the compositional homogeneity of the obtained alloy, the pre-alloy button was inverted and re-melted six times using an electric current ranging from 175 A to 250 A. The alloy buttons for each composition were then weighed again to compare the initial total weight with the final weight after arc-melting.

The weight in grams for each metal was simply calculated by following equation to give the correct composition based on wt.% for a total weight of 5 (g).

$$\frac{\text{Weight\%} \times \text{total weight (g)}}{100\%}$$

Table S1 The weight of elemental metal used to form each Cu–Sn alloy composition.

Composition wt.%	Copper weight (g)	Tin weight (g)
Cu_{80}–Sn_{20}	4.04	0.97
Cu_{70}–Sn_{30}	3.50	1.48
Cu_{50}–Sn_{50}	2.55	2.44
Cu_{40}–Sn_{60}	1.91	3.09
Cu_{20}–Sn_{80}	0.92	4.07

Table S2. Summary of the used APCVD graphene growth parameters on liquid Sn to study the effect of CH_4 concentration and residence time.

P_t (mbar)	T (°C)	t (min)	H_2 (sccm)	Ar (sccm)	CH_4 (sccm)	P_{Ar} (mbar)	P_{H2} (mbar)	P_{CH4} (mbar)	τ (s)	I_{2D}/I_G	I_D/I_G
1013	1120	5	40	400	5	910.6	91.1	11.4	8.1	0.5	1.5
1013	1120	5	30	300	5	907.2	90.7	15.1	10.7	1.1	0.7
1013	1120	5	25	250	5	904.5	90.4	18.1	12.8	1.6	0.3
1013	1120	5	20	200	5	900.4	90	22.5	15.9	0.8	0.8

Table S3. APCVD graphene growth parameters on liquid Sn using different H_2 flow rates.

P_t (mbar)	T (°C)	t (min)	H_2 (sccm)	Ar (sccm)	CH_4 (sccm)	P_{H2} (mbar)	P_{Ar} (mbar)	P_{CH4} (mbar)	τ (s)	I_{2D}/I_G	I_D/I_G
1013	1120	5	40	250	5	137.3	858.5	17.2	12.2	0.6	2.2
1013	1120	5	35	250	5	122.2	873.3	17.5	12.4	1.2	0.6
1013	1120	5	30	250	5	106.6	888.6	17.8	12.6	1.3	0.5
1013	1120	5	25	250	5	90.4	904.5	18.1	12.8	1.6	0.3
1013	1120	5	20	250	5	73.6	921	18.4	13.1	1.1	0.8

Table S4. APCVD graphene growth parameters on liquid Sn using different Ar flow rates.

P_t (mbar)	T (°C)	Growth time (min)	H_2 (sccm)	Ar (sccm)	CH_4 (sccm)	P_{H2} (mbar)	P_{Ar} (mbar)	P_{CH4} (mbar)	τ (s)	I_{2D}/I_G	I_D/I_G
1013	1120	5	25	100	5	194.8	779.2	39	27.7	0.0	1.0
1013	1120	5	25	250	5	90.4	904.5	18.0	12.8	1.6	0.3
1013	1120	5	25	300	5	76.7	920.9	15.3	10.9	1.4	0.6
1013	1120	5	25	350	5	66.6	933.0	13.3	9.5	1.3	0.6
1013	1120	5	25	400	5	58.9	942.3	11.8	8.4	0.6	1.6
1013	1120	5	25	450	5	52.8	949.7	10.5	7.5	0.6	2.1

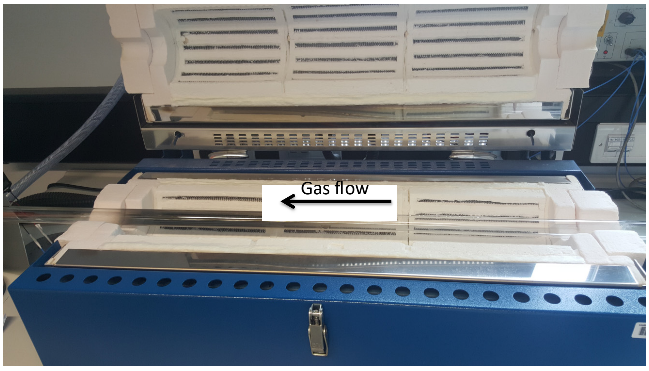

Figure S1. Photograph of the CVD reaction tube showing the change in colour of the tube's inner walls turning to black due to saturation of carbon species after using growth temperature 1140 °C for 5 min.

Table S5 Optimised growth parameters for APCVD graphene on liquid Sn.

P_t (mbar)	T (°C)	t (min.)	$Ar:H_2:CH_4$ (sccm)	P_{H2} (mbar)	P_{CH4} (mbar)	P_{Ar} (mbar)	τ (s)
1013	1120	5	250:25:5	90.4	18.1	904.5	12.85

Table S6. EDX compositional analysis of samples after APCVD graphene growth, in order to make comparisons with the initial compositions.

Cu–Sn Alloys	Cu wt.%	Sn wt.%	C wt.%	Total
$Cu_{80\%}$–$Sn_{20\%}$	82.0	17.9	0.1	100%
$Cu_{70\%}$–$Sn_{30\%}$	70.9	29.0	0.1	100%
$Cu_{50\%}$–$Sn_{50\%}$	52.7	46.7	0.6	100%
$Cu_{40\%}$–$Sn_{60\%}$	42.7	55.5	1.8	100%
$Cu_{20\%}$–$Sn_{80\%}$	13.3	77.7	9.0	100%

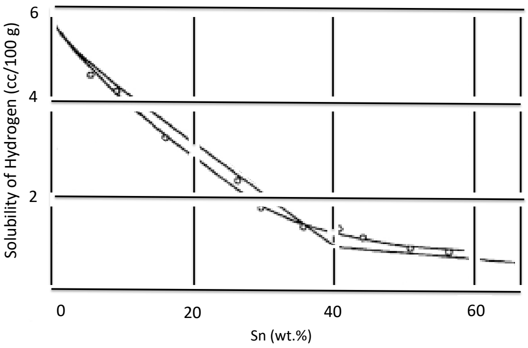

Figure S2. Solubility of hydrogen in liquid Cu–Sn alloys as a function of Sn wt.% at 1100 °C. (Reproduced with permission from [1]; Copyright The Japan Institute of Metals, 1970).

References

1. Kato, E.; Ueno, H.; Orimo, T. Solubility of Hydrogen in Liquid Copper Alloys. *Materials Transactions, JIM* **1970**, *11*, 351–358, doi:10.2320/matertrans1960.11.351.
